# Transgenic Expression of IL15 Retains CD123-Redirected T Cells in a Less Differentiated State Resulting in Improved Anti-AML Activity in Autologous AML PDX Models

**DOI:** 10.3389/fimmu.2022.880108

**Published:** 2022-05-09

**Authors:** Hong Mu-Mosley, Lauren Ostermann, Muharrem Muftuoglu, Abishek Vaidya, Challice L. Bonifant, Mireya Paulina Velasquez, Stephen Gottschalk, Michael Andreeff

**Affiliations:** ^1^ Section of Molecular Hematology and Therapy, Department of Leukemia, University of Texas MD Anderson Cancer Center, Houston, TX, United States; ^2^ Department of Bone Marrow Transplantation & Cellular Therapy, St. Jude Children’s Research Hospital, Memphis, TN, United States; ^3^ Department of Oncology, Sidney Kimmel Comprehensive Cancer Center Johns Hopkins University, School of Medicine Baltimore, MD, United States

**Keywords:** AML, T cells, immunotherapy, IL15, CD123, BiTE

## Abstract

Immunotherapy with T-cells expressing bispecific T-cell engagers (ENG T-cells) is a promising approach to improve the outcomes for patients with recurrent/refractory acute myeloid leukemia (AML). However, similar to T-cells expressing chimeric antigen receptors (CARs), their antitumor activity is limited in the setting of chronic antigen stimulation. We therefore set out to explore whether transgenic expression of IL15 improves the effector function of ENG T-cells targeting CD123-positive AML. T-cells expressing CD123-specific ENG (CD123-ENG) ± IL15 were generated by retroviral transduction from peripheral blood T cells from healthy donors or patients with AML. In this study, we characterized in detail the phenotype and effector functions of ENG T-cell populations *in vitro* and *in vivo*. IL15-expressing CD123-ENG (CD123-ENG.IL15) T-cells retained their antigen-specificity and effector function in the setting of chronic antigen exposure for more 30 days of coculture with AML blasts in contrast to CD123-ENG T-cells, whose effector function rapidly eroded. Furthermore, CD123-ENG.IL15 T-cells remained in a less differentiated state as judged by a high frequency of naïve/memory stem T-cell-like cells (CD45RA^+^CCR7^+^/CD45RO^−^CD62L^+^ cells) without evidence of T-cell exhaustion. Single cell cytokine profiling using IsoPlexis revealed enhanced T-cell polyfunctionality of CD123-ENG.IL15 T-cells as judged by effector cytokine production, including, granzyme B, IFN-γ, MIP-1α, perforin, TNF-α, and TNF-β. *In vivo*, CD123-ENG.IL15 T-cells exhibited superior antigen-specific anti-AML activity and T-cell persistence in both peripheral blood and tissues (BM, spleens, and livers), resulting in a significant survival advantage in one AML xenograft model and two autologous AML PDX models. In conclusion, we demonstrate here that the expansion, persistence, and anti-AML activity of CD123-ENG T-cells can be significantly improved by transgenic expression of IL15, which promotes a naïve/TSCM-like phenotype. However, we also highlight that targeting a single tumor antigen (CD123) can lead to immune escape, reinforcing the need to develop approaches to target multiple antigens. Likewise, our study demonstrates that it is feasible to evaluate autologous T cells in AML PDX models, which will be critical for future preclinical evaluations of next generation AML-redirected T-cell therapies.

## Introduction

The outcome of recurrent/refractory acute myeloid leukemia (AML) remains poor, and novel therapeutic approaches are urgently needed (1). T-cells expressing chimeric antigen receptors (CARs) have striking clinical activity against hematological malignancies, namely, B-cell lineage acute lymphoblastic leukemia (ALL), lymphoma, and multiple myeloma (2). However, the clinical experience with CAR T-cells for AML has been limited (3).

In addition to CARs, other molecules have been expressed in T cells to render them AML-specific, namely, WT1-specific αβ T-cell receptors (TCRs) or bispecific T-cell engagers (BiTEs, ENG T-cells) (4). In previous studies, we have shown that ENG T-cells have potent anti-tumor activity targeting CD123, tumor-associated antigens that are expressed in a broad range of AML subtypes and not only on bulk AML blasts but also on leukemia stem cells (LSCs) (5). ENG T-cells that target CD123 or other tumor antigens not only kill tumor cells directly, but also recruit bystander T-cells to tumor cells in an antigen-specific manner (5–7). This may amplify antitumor effects compared to CAR T cells that lack the ability to redirect bystander T-cells to tumor cells. More recently, investigators have also combined the expression of BiTEs and CARs in T cells to mitigate the risk of immune escape (8). However, similar to CAR T-cells, the effector function of ENG T-cells is limited in the setting of chronic antigen exposure (9). Several approaches are actively being pursued to improve the effector function of tumor-specific T-cells, namely, the transgenic expression of cytokines or the deletion of negative regulators that limit T-cell function. We and others have focused on transgenic expression of IL15, a γ-cytokine, in CAR T-cells to improve their effector function and have demonstrated that IL15 retains CAR T-cells in a less differentiated state and improves their expansion and persistence in preclinical xenograft models (10–13).

At present, there are limited data if a second genetic modification improves the effector function of ENG T-cells (9). Additionally, ENG T-cells have not been evaluated in the autologous setting in AML patient-derived xenograft (PDX) models. To address these gaps in our knowledge, we generated CD123-ENG T-cells that secrete IL15 (CD123-ENG.IL15 T-cells) and compared their effector function to unmodified CD123-ENG T-cells *in vitro* and *in vivo*. CD123-ENG.IL15 T-cells had superior anti-AML activity in repeat stimulation assays that mimic chronic antigen exposure exhibited a less differentiated phenotype and had improved persistence and anti-AML activity in AML xenograft and autologous PDX models.

## Materials and Methods

### Cells and Culture Conditions

MOLM-13 cells were purchased from the German Collection of Microorganisms and Cell Cultures GmbH (DSMZ, Braunschweig, Germany), and 293T-cells were purchased from the American Type Culture Collection (ATCC, Manassas, VA). K562 cells expressing CD123 (K562.CD123) and K562 parental cells were a generous gift from Dr. John DiPersio (Washington University School of Medicine, St. Louis, MO), and CD123 expression was evaluated by flow cytometry (**Figure S1**). Cells were validated by short tandem repeat DNA fingerprinting using the Amp-FlSTR Identifier kit according to the instructions of the manufacturer (Applied Biosystems, Foster City, CA), and were also tested to be free of mycoplasma. Cells were cultured in RPMI or DMEM complete medium (RPMI-1640 or DMEM supplemented with 10% FBS, 10 mmol/L L-glutamine, 100 U/ml penicillin, and 10 mg/ml streptomycin, Invitrogen, CA) and maintained in a humidified atmosphere containing 5% CO_2_ at 37°C.

### Construction of Retroviral Vectors

**Figure 1:**
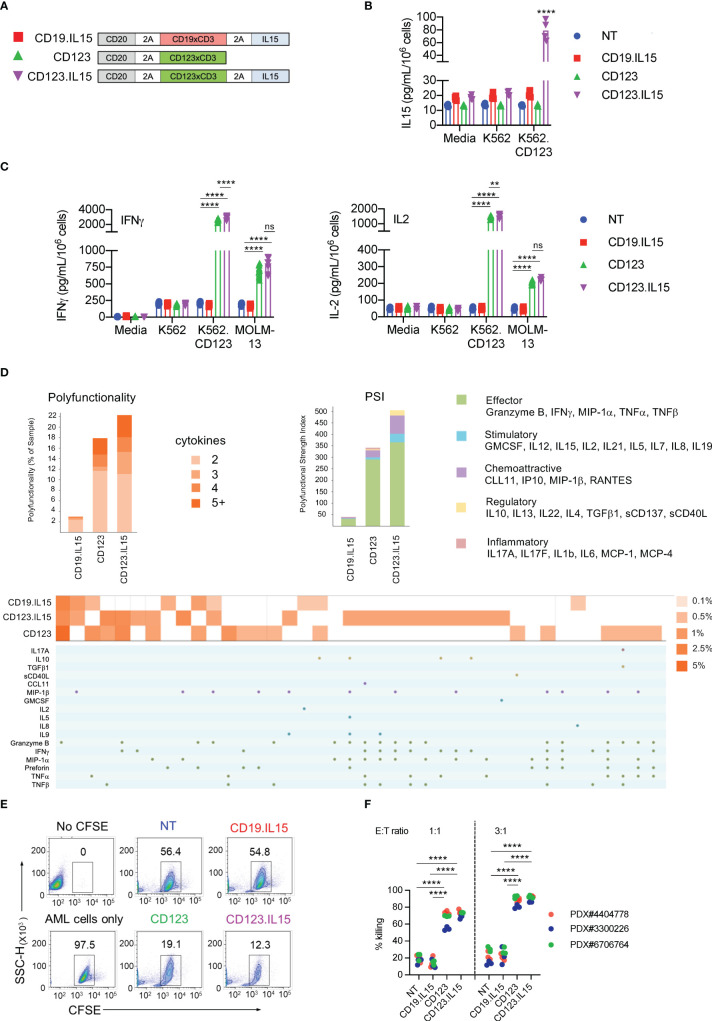
Characterization of CD123-ENG T-cells expressing IL15. **(A)** Schematic of retroviral vector constructs encoding CD123-ENG.IL15, CD123-ENG, and CD19-ENG.IL15. **(B)** IL15 concentration (pg/ml/10^6^ cells) determined by ELISA at baseline and after co-culture for 24 h with K562.CD123 or K562 at an E:T ratio of 2:1; n = 4; technical duplicates; for K562.CD123: NT, CD19.IL15, or CD123 vs CD123.IL15: ****:p <0.0001; two-way ANOVA. **(C)** IFNγ and IL2 concentration (pg/ml/10^6^ cells) determined by ELISA at baseline and after co-culture for 24 h with indicated target cells or media at an E:T ratio of 2:1; n = 4; technical duplicates; ****: p <0.0001; **: p <0.01; ns, not significant; two-way ANOVA. **(D)** CD123-ENG.IL15, CD123-ENG or CD19-ENG.IL15 T-cells were stimulated with MOLM-13 AML cells and after 20 h subjected to single-cell multiplex cytokine analysis. Left panel: T-cell polyfunctionality is shown by the percentage of each subset of polyfunctional T cells in total based on the number of cytokines. Right panel: Polyfunctional Strength Index (PSI) generated using IsoSpeak software based on cytokine strength in different categories and T cell polyfunctionality. Bottom panel: The percentage of polyfunctional T cell subsets with distinct combinatorial cytokine secretions are shown in heat map and cytokine panel. **(E, F)** Flow cytometry-based cytotoxicity assay with primary AML blast at an E:T ratio of 3:1 for 6h. **(E)** Representative flow cytometry plots for AML PDX#3300226. **(F)** Summary data, ****: p <0.0001, two-way ANOVA.

The construction of the retroviral vectors encoding the CD123-specific (scFv 26292) engager molecule or CD19-specific engager molecule, a 2A sequence, and CD20 has been described previously (5). To generate IL15-expressing ENG T-cells, we modified our existing SFG retroviral encoding CD20.CD123-ENG or CD20.CD19-ENG genes by removing the stop codon, and inserting a 2A sequence followed by the human IL15 full-length cDNA. Thus, the final expression cassette, which is driven by the long terminal repeat (LTR) promoter/enhancer of the SFG retroviral vector, contains three transgenes that are separated by 2A sequences (**Figure 1A**). RD114-pseudotyped retroviral particles were generated by transient transfection of 293T-cells as described previously (5).

### Generation of IL15 Expressing ENG T-Cells

Human peripheral blood mononuclear cells (PBMC) from healthy donors were purchased from the Gulf Coast Blood Center (Houston, TX). Genetically modified T cells were generated from PBMCs as previously described (5, 7). Briefly, T cells were activated by stimulation on OKT3 (CRL-8001, ATCC) and anti-CD28 (Becton Dickinson, Mountain View, CA) coated non-tissue culture treated 24-well plates. Recombinant human interleukin (IL)7 (10 ng/μl, R&D Systems, Minneapolis, MN) and IL15 (5 ng/μl, R&D Systems) were added to cultures on day 2, and the following day, cells were transduced with retroviral particles immobilized on RetroNectin (Clontech Laborotories, Inc., Mountain View, CA). T cells were maintained and expanded in the presence of IL7 and IL15. Cells were analyzed for expression of CD20 by flow cytometry on 5 to 7 days post transduction (5) and T cells with ≥35% transduction efficiency were used for experiments on 7–12 days post transduction.

### Flow Cytometry

Fluorochrome-conjugated isotype controls, anti-CD123, anti-CD3, anti-CD4, anti-CD8, anti-CD33, anti-CD45, anti-CD45RA, anti-CD62L, anti-CCR7, anti-PD-1, anti-TIM3, anti-LAG3, anti-Annexin-V, 7-AAD, DAPI, and anti-mouse CD45 were purchased from BD Biosciences or BioLegend (San Diego, CA). Analysis was performed on at least 20,000 cells per sample using a LSR II flow cytometer (BD Biosciences) and a Gallios Flow Cytometer, and analyzed using Kaluza Analysis Software (Beckman Coulter, Indianapolis, IN).

### Cytokine Secretion Assay

ENG T-cells were plated with target cells at a 2:1 ratio. Following 24 h of culture, the supernatant was harvested and analyzed for the presence of interferon (IFN)γ, IL2, or IL15 using ELISA kits (R&D systems) according to the instructions of the manufacturer.

### Long-Term Coculture With Repeated Stimulations

ENG T-cells were maintained in culture and stimulated every five days with MOLM-13 cells (E:T ratio 1:1) without any addition of exogenous cytokines. Cells were cultured for up to 40 days, and viable T- and MOLM-13 cells were counted using counting beads and analyzed by flow cytometry every five days before the addition of fresh MOLM-13 cells. Cells are sub-cultured to prevent overgrowth in co-cultures.

### Luciferase-Based Cytotoxicity Assay

Firefly-luciferase expressing K562 parental and K562.CD123 cells were generated by transduction with a lentiviral vector encoding firefly luciferase and a mCherry fluorescent protein/puromycin resistance gene (mCherrypuro; pCDH.CMV.CD123.EF1.mCherrypuro) and mCherry-positive K562 parental or K562.CD123 cells were sorted by flow cytometry and maintained in RPMI complete medium. Luciferase-based cytotoxicity assay of ENG T-cells against target cells was performed using a modified protocol as previously reported (14). Briefly, ENG T-cells were plated with target cells at a 3:1 ratio. After 20 h, the luciferase activity of the cells was measured by a luminescence microplate reader (Molecular Devices, San Jose, CA) after adding D-luciferin following the protocol of the manufacturer (Promega, Madison, WI). The mean percentage of cytotoxicity was calculated based on the total flux [p/s] relative to the leukemia cells only control, and the mean percentage of cytotoxicity is shown as 100 ∗ (mean [p/s] of leukemia cell only control − mean [p/s] of the experimental group)/(mean [p/s] of leukemia cell only control).

### Primary AML Blasts Cytotoxicity Assay

Under informed written consent, bone marrow or peripheral blood samples were obtained from adult patients diagnosed with AML (**Table 1**) during routine diagnostic workup in accordance with regulations and protocols approved by the Institutional Review Board (IRB) Committee of The University of Texas MD Anderson Cancer Center. Mononuclear cells were separated by Ficoll–Hypaque (Sigma Chemical Co.) density-gradient centrifugation and CD123 expression was evaluated by flow cytometry (**Figure S1**). After T cells were removed using CD3 magnetic beads (Miltenyi Biotec Inc. Auburn, CA) mononuclear cells were cryopreserved in liquid nitrogen.

**Table 1:** Primary CD123-positive AML patient samples.

AML#	Number of previous therapies	ENG T-cells	AML PDX	Site	Mutations	Cytogenetics
4404778	4	No	Yes	PB	FLT3-ITD, DNMT3A, IDH1, NPM1 [CMS28]	46,XX [20]
3300226	1	No	Yes	PB	Not detected	44,X,add(X)(p22.1),-2,-4,del(5)(q15q33),del(11)(p13),-12,-13,-13,-17,17,+21,add(22)(p11.2),+4mar[9]//46,XY [11]
6706764	0	Yes	No	BM	ASXL1, CEBPA, SRSF2, EZH2	45,X,idic(X)(q13),t(1;7)(q21;q22),-7,del(20)(q11.2q13.3)[19]/46,XX [1]
6697688	3	Yes	Yes	PB	ETV6, U2AF1, FLT3, WT1	46,XY,der(6)t(6;9)(p23;q34),der(9)add(9)(p22)del (9)(q13q34)t(6;9),-10,+mar[17]/46,XY, t(6;9) (p23;q34) [cp3]
6701348	0	Yes	Yes	PB	NRAS, KIT, FLT3	46,XY,der(7)t(7;8)(q22;q13),inv(16)(p13.1q22)[20]

The cytolytic activity of ENG T-cells against primary AML blasts was analyzed using the 7-AAD/CFSE cell-mediated cytotoxicity assay following the instructions of the manufacturer (Cayman Chemical, Ann Arbor, MI) (15). Briefly, AML mononuclear cells were thawed and then labeled with carboxyfluorescein diacetate succinimidyl ester (CFSE) before co-culture with ENG or NT T-cells at ratios of 3:1 and 1:1 in RPMI complete medium. After 6 h, cells were enumerated by flow cytometry using counting beads. Dead cells were stained by 7-aminoactinomycin D (7-AA). The surviving AML cells were identified as 7-AAD^−^CFSE^+^ cells by FACS. The mean percentage of cytolytic activity in each experiment was calculated as 100 ∗ (#AML cells only control − #experimental group)/(#AML cell only control).

### Single-Cell Multiplex Cytokine Profiling of ENG T-Cells

On day 7 post retroviral transduction, 10^7^ bulk ENG T-cells (NT, CD19-ENG.IL15, CD123-ENG, or CD123-ENG.IL15, transduction efficiency ~50%) were mixed and co-cultured with MOLM-13 cells at an E:T ratio 1:1 in RPMI complete media. After 20 h, T cells were enriched by the removal of MOLM-13 cells using anti-CD33 and anti-CD123 magnetic beads (Miltenyi). After the removal of residual magnetic beads, transduced and NT T-cells were separated from enriched T-cells using anti-CD20 magnetic beads (Miltenyi). Transduced and NT T-cells were then stained with stain A (nonspecific cell membrane violet, IsoPlexis) and Alexa Fluor 647-conjugated anti-human CD4 (IsoPlexis, Branford, CT) before loading onto IsoCode chips. Each IsoCode chip contains ~12,000 microchambers pre-patterned with a full copy of a 32-plex antibody array including effector: granzyme B, TNFα, IFN-γ, MIP1α, perforin, TNFβ; stimulatory: GM-CSF, IL2, IL5, IL7, IL8, IL9, IL12, IL15, IL21; chemoattractant: CCL11, IP-10, MIP-1β, RANTES; regulatory: IL4, IL10, IL13, IL22, sCD137, sCD40L, TGFβ1; and inflammatory: IL6, IL17A, IL17F, MCP-1, MCP-4, IL1β. Cytokines secreted were captured by IsoLight at the single-cell level, and the polyfunctional profile (≥2 proteins per cell) of single cells was evaluated by IsoSpeak software.

### Xenograft AML Mouse Model

All animal studies were conducted in accordance with the guidelines and protocols approved by the Institutional Animal Care and Use Committees at The University of Texas MD Anderson Cancer Center. To establish the MOLM-13 xenograft AML model, (NOD.Cg-Prkdcscid Il2rgtm1Wjl Tg(CMV-IL3,CSF2,KITLG)1Eav/MloySzJ, NSGS) mice (8–12-week old, both female and male, The Jackson Laboratory, Bar Harbor, MA) were intravenously injected with 1.5 × 10^5^ MOLM-13 cells, genetically modified to express a GFP firefly luciferase fusion gene (MOLM-13.GFP.ffLuc) (5), and randomly divided into four groups (NT, CD19-ENG.IL15, CD123-ENG, or CD123-ENG.IL15). Mice were infused with 10^7^ T-cells per mouse of each type on day 2 post MOLM-13 injection. As described previously, bioluminescence imaging (BLI) was used to monitor tumor burden (16).

For primary AML patient-derived xenograft (PDX) models, NSGS mice were sub-lethally irradiated (250 cGy) the day before intravenous injection of 2 × 10^6^ patient-derived AML cells (PDX#6697688 or PDX#6701348). After engraftment was confirmed, mice were randomly divided into groups to receive one dose of T-cell infusion or PBS as an untreated control. T-cells and AML cell growth *in vivo* were monitored by flow cytometry of peripheral blood or mouse tissues. The AML xenograft mice were then followed up for survival.

### Mass Cytometry

Cells from murine bone marrow and spleens were barcoded and pooled before surface staining with a panel of 52 antibodies (metal isotope-conjugated, **Table S1**) and were analyzed on a Helios mass cytometer (Fluidigm) as reported previously (17, 18). Using a cloud-based computational platform OMIQ.ai (Omiq, Inc. Santa Clara, CA), data were normalized and analyzed with gating on CD45^+^ cells. Equal sampling of 65,143 events per sample and a total of 378,858 cells from 4 samples/groups as a dataset were analyzed using Uniform Manifold Approximation and Projection (UMAP) and PhenoGraph clustering analysis. The distribution and expression characteristics of exhaustion and phenotypic markers on human CD3^+^ T-cells were analyzed and compared across all samples.

### Statistical Analyses

GraphPad Prism 8 software (GraphPad software, Inc., San Diego, CA) was used for statistical analysis. Measurement data were presented as mean ± standard error (SE). A two-tailed *t*-test was used for comparison between two groups. For comparisons of three or more groups, the values were analyzed by one-way or two-way ANOVA with Bonferroni’s post-test. For the mouse experiments, survival, determined from the time of T-cell infusion, was analyzed by the Kaplan–Meier method and by the log-rank Mantel–Cox test. P-values of less than 0.05 were considered statistically significant.

### Data Sharing Statement

For data sharing, contact the corresponding authors at mandreef@mdanderson.org or stephen.gottschalk@stjude.org.

## Results

### Generation of IL15 Expressing CD123-ENG T-Cells

To generate IL15-expressing ENG T-cells, we created retroviral vectors encoding CD20 as a safety switch, CD123-ENG or CD19-ENG (control), and IL15 separated by a 2A sequence (CD123-ENG.IL15 and CD19-ENG.IL15, **Figure 1A**). ENG T-cells were generated from PBMCs of healthy donors by retroviral transduction and expanded as previously described (5). Similar transduction efficiencies were achieved as judged by flow cytometric analysis for CD20 (**Figure S2A**, mean: 70.7%, n = 7), and ENG T-cells had a similar immunophenotype and growth rate compared to non-transduced (NT) T-cells (**Figures S2B, C**). To confirm the IL15 production by ENG T-cells, we performed an ELISA pre- and post-coculture with K562.CD123 cells. While CD123-ENG.IL15 and CD19-ENG.IL15 T-cells produced low levels (mean: 12.7 pg/ml) of IL15 at baseline, there was a significant increase in IL15 production (mean: 70.8 pg/ml) by CD123-ENG.IL15 T-cells post antigen-specific activation (**Figure 1B**).

### CD123-ENG.IL15 T-cells recognize AML cell lines and primary AML blasts in an antigen-dependent fashion

We performed coculture assays with CD123-positive (K562.CD123, MOLM-13; **Figure S1**) and CD123-negative (K562; **Figure S1**) target cells. CD123-ENG and CD123-ENG.IL15 T-cells produced significant amounts (p <0.001) of IFNγ and IL2 only in the presence of K562.CD123 and MOLM-13 compared to non-transduced (NT) and CD19-ENG.IL15 T-cells, demonstrating antigen-specificity (**Figure 1C**). Additionally, CD123-ENG.IL15 T-cells secreted significantly (p <0.05) higher amounts of IFNγ and IL2 than CD123-ENG T-cells in the presence of K562.CD123 (**Figure 1C**). We also performed IsoPlexis single-cell proteomics analysis (19) to determine cytokine production and polyfunctionality of ENG T-cells post antigen-specific activation with MOLM-13 cells. A higher percentage of CD123-ENG.IL15 T-cells produced multiple cytokines compared to CD123-ENG T-cells, resulting in a higher polyfunctional strength index (PSI, **Figure 1D**). Cytokine profiles further indicated that CD123-ENG.IL15 T-cells secreted a broader array of immuno-stimulatory molecules/cytokines (e.g., Granzyme B, IFN-γ, MIP-1a, TNF-α, TNF-β) than CD123-ENG T-cells (**Figure 1D** bottom), indicating improved polyfunctionality.

Specificity was confirmed in luciferase-based cytotoxicity assays in which CD123-ENG.IL15 T-cells demonstrated enhanced (p <0.05) specific cytotoxic activity against K562.CD123 target cells compared to CD123-ENG T-cells (**Figure S2D**). To demonstrate that CD123-ENG and CD123-ENG.IL15 T-cells kill primary CD123-positive AML blasts, we performed CFSE/7-AAD cell-based cytotoxicity assays with 3 primary AML samples. Both, CD123-ENG and CD123-ENG.IL15 T-cells demonstrated significant (p <0.001) cytotoxicity against CD123-positive primary AML blasts compared to NT or CD19-ENG.IL15 T-cells (**Figures 1E, F**).

### CD123-ENG.IL15 T-Cells Retain Their Anti-Leukemia Activity in Repeat Stimulation Assays That Mimic Chronic Antigen Exposure

**Figure 2:**
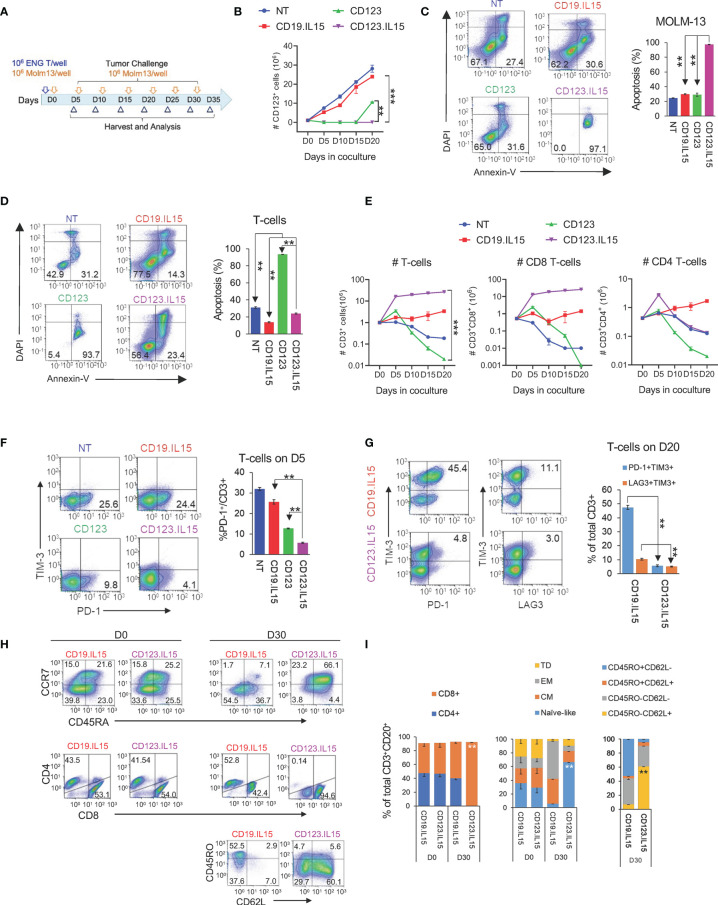
Transgenic Expression of IL15 improves effector function of CD123-ENG T-cells. **(A)** Scheme of repeat stimulation assay. AML cells and T cells in co-cultures were enumerated using counting beads by flow cytometry prior to each re-challenge. **(B)** CD123^+^ MOLM-13 AML cell count from the repeat stimulation assay prior to the 2nd (D5), 3rd (D10), 4th (D15), and 5th (D20) stimulation. **(C, D)** Apoptosis measured by Annexin-V^+^DAPI^-^ flow cytometry **(C)** of AML and **(D)** T-cells on day 20. Representative flow cytometry plots and summary data are shown. **(E)** CD3^+^, CD8^+^, and CD4^+^, T-cell counts (log10) in coculture assay overtime. **(F)** PD-1 and TIM3 expression in T-cells on day 5. Left panel: representative flow cytometry plots gated on CD3^+^ T-cells. Right: summary data. **(G)** Expression PD-1, TIM3, and LAG-3 in T-cells on day 20. Left panel: representative flow cytometry plots gated on CD3^+^ T-cells. Right: summary data. **(H, I)** Phenotypic analysis on CD3^+^CD20^+^ of CD123.IL15 and CD19.IL15 T-cells before and on day 30 of co-culture. **(H)** Representative flow cytometry plots. **(I)** Summary data, n = 3 for all data shown in panels; data are shown as mean ± SE, ***:p <0.001, **:p <0.01, *t*-test.

Next, we carried out experiments to determine whether IL15 expression enhances the effector function of CD123-ENG T-cells in repeat stimulation assays that mimic chronic antigen exposure. Briefly, ENG T-cells were co-cultured with MOLM-13 AML cells at an E:T ratio of 1:1, and fresh MOLM-13 cells were added to the co-culture every 5 days (**Figure 2A**). Before adding fresh tumor cells, live total CD123-positive MOLM-13 and CD3-positive T-cells were enumerated by flow cytometric analysis (gating strategy shown in **Figures S3A, B**). For the first three stimulations, CD123-ENG and CD123-ENG.IL15 T-cells had comparable killing activity (**Figure 2B**). However, on day 20, after the fourth stimulation, most of the AML cells were alive in the presence of CD123-ENG T-cells (**Figure 2C**). CD123-ENG T-cells had also undergone apoptosis in contrast to CD123-ENG.IL15 T-cells (**Figure 2D**). On day 40, following eight stimulations, CD123-ENG.IL15 T-cells maintained their cytolytic activity (**Figure S3C**). Improved cytolytic activity of CD123-ENG.IL15 T-cells was mirrored by T-cell expansion, particularly of the CD8-positive T-cell compartment (**Figure 2E**), compared to other effector T-cell populations.

To gain insight into the mechanism of improved effector function of CD123-ENG.IL15 T-cells, we focused on determining markers of T-cell exhaustion (PD-1, TIM-3, and LAG3), and performed T-cell subset analysis. After the first stimulation (day 5), CD123-ENG.IL15 T-cells expressed significantly lower levels of PD-1 compared to CD123-ENG, CD19-ENG.IL15, and NT control T-cells, in particular in the CD4-positive T-cell compartment (**Figures 2F**; **S3D**). After the fourth stimulation (day 20), the frequency of double-positive (PD-1/TIM-3 or LAG3/TIM-3) CD123-ENG.IL15 T-cells was significantly lower compared to CD19-ENG.IL15 T-cells, consistent with a less exhausted phenotype (**Figure 2G**). Since >98% of CD123-ENG T-cells had died by day 20 (**Figure 2E**), it was impossible to perform this type of analysis for this effector T-cell population. Comparing the immune phenotype of CD123-ENG.IL15 T-cells before the first (day 0) and after the sixth stimulation (day 30) revealed a significant enrichment of CD8-positive T-cells (**Figures 2H, I**). Additionally, T cells maintained a high percentage of CD45RA^+^CCR7^+^/CD45RO^−^CD62L^+^ cells, consistent with a naïve-like/memory stem T-cell (TSCM)-like phenotype.

### Transgenic Expression of IL15 in CD123-ENG T-Cells Improves Persistence and Anti-Leukemia Activity Resulting in Improved Survival in AML Xenograft Model

**Figure 3:**
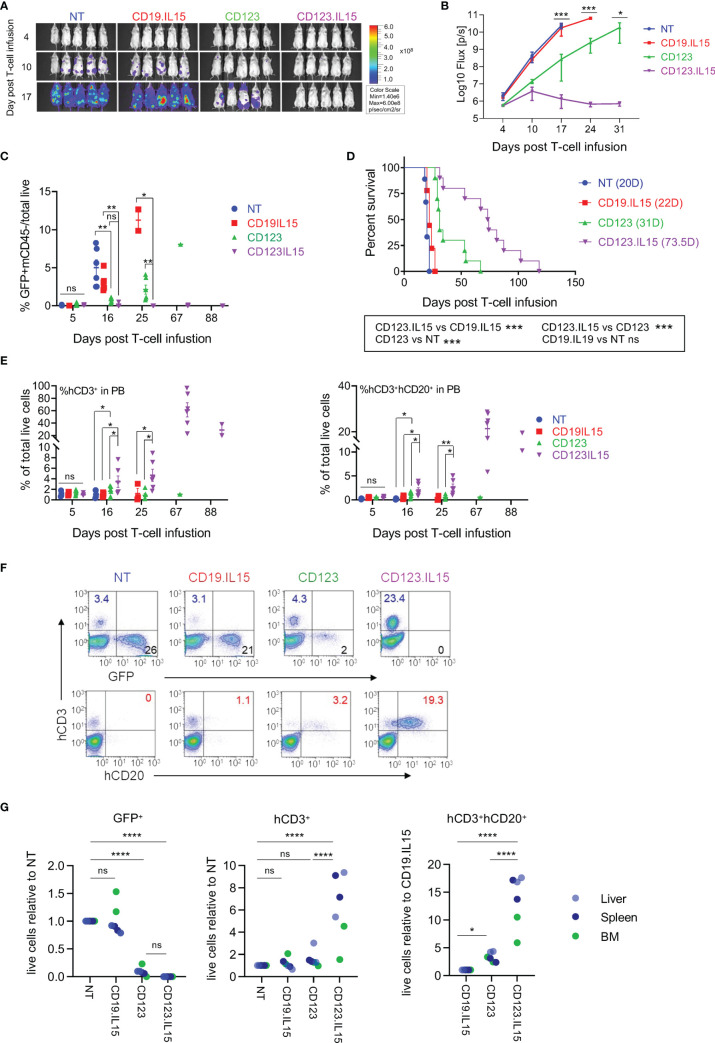
IL15-expression in CD123-ENG T-cells results in enhanced persistence and improved anti-leukemia efficacy in AML xenograft model. MOLM-13.GFP.ffluc-bearing NSGS mice received a single dose of 10^7^ CD123-ENG.IL15, CD123-ENG, NT, or CD19-ENG.IL15 T-cells per mouse. MOLM-13 leukemia growth was measured by serial bioluminescence imaging (n = 9 to 11 mice per group). **(A)** Representative bioluminescence images. **(B)** Quantitative bioluminescence data. **(C)** Circulating number of GFP^+^/mCD45^-^/total live MOLM-13 cells determined by flow cytometry analysis post T-cell infusion; data are shown as mean ± SE, ***: p <0.001, **: p <0.01, *: p <0.05, ns: not significant, *t*-test. **(D)** Kaplan–Meier survival analysis, ***: p <0.001, ns, non-significant, two-tailed log-rank Mantel–Cox test. **(E)** Circulating number of hCD3^+^mCD45^−^ (left panel) and hCD3^+^hCD20^+^mCD45^−^ T-cells determined by flow cytometry analysis post T-cell infusion; data are shown as mean ± SE, **: p <0.01, *: p <0.05, ns, not significant, student *t*-test. **(F, G)** On day 20 two mice were euthanized of each group and the frequency of GFP^+^mCD45^−^ MOLM-13 AML, hCD3^+^mCD45^−^ T-cells, and hCD3^+^hCD20^+^mCD45^−^ T-cells in liver, spleen, and bone marrow (BM) was determined. **(F)** Presentative flow cytometry analysis of spleen sample. **(G)** Relative frequency of GFP^+^mCD45^−^ MOLM-13 AML, hCD3^+^mCD45^−^ T-cells, and hCD3^+^hCD20^+^mCD45^−^ T-cells in analyzed tissue, ****: p <0.0001, ns, not significant, two-way ANOVA.

We next evaluated the anti-leukemic activity and persistence of CD123-ENG.IL15 T-cells in the MOLM-13 AML xenograft mouse model. MOLM-13.GFP.ffLuc-bearing NSGS mice (n = 47, 20 F and 27 M) received a single dose of T cells (NT, CD19-ENG.IL15, CD123-ENG, or CD123-ENG.IL15; 5F and 6–7 M each group); their phenotype is shown in **Figure S4**. CD123-ENG.IL15 and CD123-ENG T-cells had significant anti-AML activity compared with NT or CD19-ENG.IL15 T-cells within 10 days after T-cell infusion as judged by bioluminescence imaging (BLI, **Figures 3A, B**). However, only CD123-ENG.IL15 T-cells could control leukemia progression for >25 days as judged by BLI and by flow cytometry for GFP-positive AML cells in the peripheral blood of the mice (**Figure 3C**). This improved anti-AML activity translated into a significant survival advantage compared to all other treatment groups (**Figure 3D**). Mice that received CD123-ENG.IL15 T-cells most likely died of xenogeneic graft-versus-host disease (GVHD), which is commonly seen in NSG mouse models >50 days post infusion of human T cells (20–22). GVHD is also supported by the fact that mice had high levels of circulating T cells at days 67 and 88 post T-cell infusion (**Figure 3E**) as described in the next paragraph.

To get insight into the *in vivo* fate of T-cells, we performed flow cytometry of human T cells in mouse peripheral blood (% hCD3^+^mCD45^−^/total live cells). On day 5 post T-cell infusion, the frequency of circulating human CD3-positive T-cells was similar among all treatment groups (1.1%; **Figure 3E**). Subsequently, we observed a significant, sustained expansion of CD3-positive T-cells only in mice that had received CD123-ENG.IL15 T-cells. These cells were genetically modified since they expressed human CD20, which is encoded by the CD123-ENG.IL15 retroviral vector (**Figure 3E**). To obtain additional insights into the distribution of human T and leukemic cells, 2 mice from each treatment group were euthanized on day 20 post T-cell infusion to analyze the BM, spleen, and liver for the presence of leukemia and human T cells. There was a striking reduction in the leukemia burden in the liver, spleen, and BM of mice treated with CD123-ENG or CD123-ENG.IL15 T-cells compared to mice that had received NT or CD19-ENG.IL15 T-cells (**Figures 3F, G**; **S5**). Similar to our findings in peripheral blood (**Figure 3E**), the mice treated with CD123-ENG.IL15 T-cells also had a significantly decreased leukemia burden and a greater frequency of human T-cells (total and CD123-ENG.IL15 T-cells) in the liver, spleen, and BM compared to other treatment groups (**Figures 3F, G**; **S5**).

### Autologous, Patient Derived CD123-ENG.IL15 T-Cells Demonstrate Enhanced Persistence and Anti-Leukemia Activity in AML PDX Models

**Figure 4:**
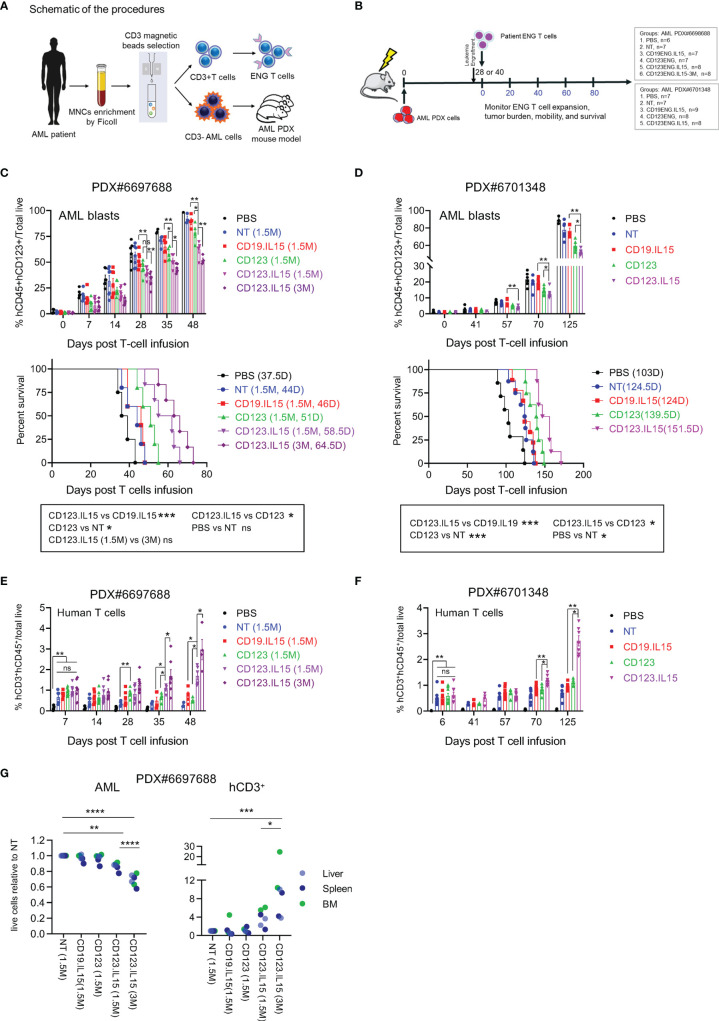
Autologous CD123-ENG.IL15 T-cells have improved persistence and anti-leukemia activity in AML PDX mouse models. **(A)** Schema of generation matching CD123-ENG.IL15 T-cells and AML PDX mouse model. **(B)** Schema of AML PDX#6698688 and PDX#6701348 animal experiments. **(C)** PDX#6698688 AML PDX model: Circulating hCD45^+^hCD123^+^mCD45^−^ AML PDX cells (top panel) and Kaplan–Meier survival analysis (bottom panel). **(D)** PDX#6701348 AML PDX model: Circulating hCD45^+^hCD123^+^mCD45^−^ AML PDX cells (top panel) and Kaplan–Meier survival analysis (bottom panel). Statistical analysis for in **(C, D)** data are shown as mean ± SE, ***p <0.001, **p <0.01, *p <0.05, ns: not significant, top panels: *t*-test; bottom panels: two-tailed log-rank Mantel–Cox tests. **(E, F)** Circulating hCD45^+^hCD3^+^mCD45^−^ T-cells of total live cells of mouse PB in **(E)** PDX#6698688 and **(F)** PDX#6701348 model; data are shown as mean ± SE, **p <0.01, *p <0.05, ns, not significant, *t*-test. **(G)** On day 28 post infusion two mice per group of PDX#6698688 model were euthanized and the frequency of human AML and T cells were determined. Relative frequency of human AML and T cells in analyzed tissues is shown, ****: p <0.0001, ***p <0.001, **p <0.01, *p <0.05, two-way ANOVA.

In the final set of experiments, we compared the anti-leukemia activities of autologous CD123-ENG and CD123-ENG.IL15 T-cells in AML patient-derived xenograft (PDX) models. Three primary CD123-positive AML patient samples were chosen. The CD3-negative component was injected into NSGS mice to generate AML PDX models (**Figure 4A**), whereas the CD3-positive component was used to generate ENG T-cells. ENG T-cells were generated from the three AML patient samples with similar mean transduction efficiency (~48%) and *ex vivo* expansion compared to ENG T-cells generated from healthy donors (**Figures S6A, B**). Transduction did not affect the T-cell subset composition (**Figure S6C**) either, except that ENG T-cells from patients with AML had a higher percentage of CD8-positive T-cells compared to those from healthy donors (**Figure S6D**).

PDX mouse models were successfully established for two of the three AML patient samples (PDX#6697688, PDX#6706348), and we evaluated the anti-leukemia efficacy of autologous ENG T-cells in both models. Once engraftment was confirmed, mice were randomized into different groups to receive one dose of T-cells (NT, CD19-ENG.IL15, CD123-ENG, or CD123-ENG.IL15) or untreated controls (**Figure 4B**). AML engraftment was confirmed on day 28 post injection for PDX#6697688 (**Figure 4C**) and on day 40 for PDX#6701348 (**Figure 4D**). For PDX#6697688 we had sufficient CD123-ENG.IL15 T-cells to inject two groups of mice with different cell doses (1.5 × 10^6^ or 3 × 10^6^ T cells per mouse) for a dose response. For PDX#6701348 we had enough cells to inject 1 × 10^7^ T cells per mouse. In both models, adoptive transfer of CD123-ENG T-cells and CD123-ENG.IL15 T-cells significantly delayed AML progression, resulting in a significant survival advantage (**Figures 4C, D**), and CD123-ENG.IL15 T-cells dose-dependently had greater antitumor activity than CD123-ENG T-cells. Longitudinal tracking of human T-cells in the mouse peripheral revealed a higher number of human T-cells in CD123-ENG.IL15 group mice compared to the other treatment groups in both PDX models (**Figures 4E, F**); circulating human T-cells were genetically modified, as judged by the expression of human CD20 (**Figures S6E, F**), which is encoded by the CD123-ENG.IL15 retroviral vector.

To determine leukemia burden and the frequency of human T-cells in the BM, liver, and spleen, we euthanized 2 mice from each treatment group for PDX#6697688 on day 28 post T-cell infusion. In each organ, there was a decreased leukemia burden and an increased frequency of human T cells in mice that had received CD123-ENG.IL15 T-cells compared to other treatment groups (**Figures 4G**; **S7**), mirroring the results of our peripheral blood analysis. Cells from murine BM and spleens were also analyzed by CyTOF. Human CD3^+^ T cells were identified as an isolated cluster in the UMAP plot (**Figures S8A, B**). CD123-ENG.IL15 T cells contained more CD45RA^+^ and CCR7^+^ T cells than CD123-ENG T cells (**Figures S8A, B**), mirroring the phenotypic analysis of our *in vitro* analysis. Furthermore, CD123-ENG.IL15 T cells were less exhausted than CD123-ENG T cells as measured by exhaustion markers, namely, LAG-3, CD39, T-bet, 2B4, PD-1, inducible co-stimulator (ICOS), and glucocorticoid-induced TNF-R-related protein (GITR; **Figures S8A, B**).

Finally, to explore the possibility of immune escape in both models, we analyzed the expression intensity of CD123 on AML blasts post T-cell infusion. While AML blasts in the peripheral blood of mice still expressed CD123 post therapy with CD123-ENG or CD123-ENG.IL15 T-cells, the surface density (estimated by mean fluorescence intensity) was lower than AML blasts from mice that had received NT or CD19-ENG.IL15 T-cells, or no therapy (**Figures S9A–C**).

## Discussion

Our studies demonstrate that transgenic expression of IL15 strikingly improves the effector function of CD123-ENG T-cells in repeat stimulation assays that mimic chronic antigen exposure. CD123-ENG.IL15 T-cells retained a naïve/TSCM-like phenotype and expressed low levels of exhaustion markers and, *in vivo*, had superior anti-AML activity compared to CD123-ENG T-cells not only in xenografts derived from established cell lines but also in those derived from leukemias of a primary patient.

Currently, there is no ideal antigen for AML-directed immunotherapy since most targets are either expressed on normal hematopoietic stem cells (e.g., CD33 or CD123) or mature neutrophils (e.g., CLL-1) (4, 23–25). We and others have focused on CD123-targeted immunotherapy since CD123 is consistently expressed not only on bulk AML blasts but also on leukemia stem cells (LSCs) (26, 27). Bispecific CD123xCD3 antibodies are currently being explored in several clinical studies. Additionally, early-phase clinical studies with CD123-CAR T-cells are in progress. We have focused on our CD123-specific T-cell approach on T cells that are genetically modified with BiTEs since these so-called ENG T-cells can redirect bystander T-cells to tumor cells in contrast to CAR T-cells (5–7).

We modified T-cells with a SFG retroviral vector encoding a CD123-ENG molecule and IL15, and observed significant but moderate IL15 production only after T-cell activation (**Figure 1B**). This observation is consistent with our and findings of other investigators (11), and is most likely due to transcription factors that are upregulated post T-cell activation and bind to the LTR promoter/enhancer of the SFG retroviral vector (28). This suggests that IL15 is preferentially produced at tumor sites at which CD123-ENG.IL15 T-cells are activated by CD123-positive AML blasts (BM or spleen). We believe that this may serve as an important safety feature since systemic administration of IL15 has been associated with toxicities in human clinical studies (29, 30). Other investigators have shown that the systemic delivery of CAR T-cells expressing IL15 is well tolerated in NSG and immune competent murine models, and clinical studies are in progress (12, 13, 31, 32). Systemic delivery of CAR NK cells expressing IL15, on the other hand, can induce toxicities in NSG mouse models (33, 34). Thus, it is likely that the safety profile of T cells expressing IL15 and NK cells expressing IL15 is different. Since the expression of CD123-ENGs is controlled by the same promoter as IL15, its expression should also be higher post activation. Indeed, we have shown this previously for other ENG molecules that were encoded by the same retroviral backbone (6). While we have previously measured ENG secretion by T cells (including CD123-ENG) with an ELISA (5, 7), we did not perform these studies here.

After the initial stimulation, we observed only minor differences concerning cytokine production and cytolytic activity between CD123-ENG and CD123-ENG.IL15 T-cells, which is consistent with other studies in which the functional benefit of a second genetic modification only becomes obvious in repeat stimulation assays that mimic chronic antigen exposure (11, 35). The transgenic expression of IL15 significantly improved the ability of CD123-ENG T-cells to recursively kill target cells. While previous studies have shown that IL15 improves the effector function of T cells expressing CARs with a signaling domain that includes a costimulatory domain, to our knowledge, only one other study has demonstrated the benefit of transgenic expression of IL15 in T cells that are solely activated through CD3z (36).

Transgenic expression of IL15 in CD123-ENG T-cells had profound effects on the phenotype of T-cells, resulting in a naïve/TSCM-like phenotype (CD45RA^+^CCR7^+^/CD45RO^−^CD62L^+^ cells) (37). Since CD95 is expressed upon T-cell activation, it was impossible to differentiate between naïve-and TSCM-like subsets (9), and we are planning to perform detailed epigenetic analyses used to accurately define T-cell subsets in the future (38). Recent studies have suggested that constitutive expression of CARs with costimulatory domains in T-cells is harmful to their T-cell phenotype and function, and investigators have devised strategies to either induce CAR expression or use small molecule inhibitors to intermittently rest CAR T-cells (39–41). Our results suggest that similar effects can be obtained using transgenic expression of IL15. Our studies corroborate findings by other investigators that transgenic expression of IL15 in CAR T-cells retains these cells in a less differentiated state (12, 42). However, our study is among the first ones to demonstrate the benefit of transgenic IL15 in the setting of chronic antigen exposure.

*In vivo*, transgenic expression of IL15 in CD123-ENG T-cells resulted in enhanced expansion, persistence, and improved anti-AML activity in AML xenograft models and in two AML PDX models. In both AML PDX models, we used autologous CD123-ENG.IL15 T-cells. To our knowledge, our study is one of the first to demonstrate the anti-AML efficacy of CD123-redirected T-cells in autologous preclinical models. Despite the improved anti-AML efficacy of CD123-ENG.IL15 T-cells, leukemia eventually progressed in both PDX models. We found decreased cell surface expression of CD123 post CD123-redirected T cell therapy in both AML PDX models, indicating that the development of antigen loss variants are one mechanism of therapeutic failure in our models, and that strategies to target multiple antigens expressed on AML blast are needed (43–45). Downregulation of antigen expression instead of antigen loss has been highlighted as an immune escape mechanism, in particular for CD22-CAR T-cell therapies (46), and the importance of antigen density for optimal CAR T cell recognition has been demonstrated in preclinical studies (PMID: 32193224) (47). Likewise, even without endogenous immune cells, characteristic of PDX mouse models, AML blasts have direct immunosuppressive effects, which could explain why T-cell therapy was not curative in our model (48). Indeed, a recent study demonstrated no anti-AML activity of CD33-CAR T-cells in ten patients with AML, highlighting that the adoptive immunotherapy of CAR T-cells for AML still faces formidable challenges (49). In this regard, our study suggests that AML PDX models are ideal for modeling autologous CAR T-cell therapy for AML, and we are planning to perform additional mechanistic studies in the future.

In conclusion, we demonstrate here that the expansion, persistence, and anti-AML activity of CD123-ENG T-cells can be significantly improved by transgenic expression of IL15, which promotes a naïve/TSCM-like phenotype. Likewise, our study demonstrates that it is feasible to evaluate autologous T-cells in AML PDX models, which will be critical for future preclinical evaluations of next generation AML-redirected T-cell therapies.

## Data Availability Statement

The raw data supporting the conclusions of this article will be made available by the authors, without undue reservation.

## Ethics Statement

The studies involving human participants were reviewed and approved by the Institutional Review Board (IRB) Committee of The University of Texas MD Anderson Cancer Center. The patients/participants provided their written informed consent to participate in this study. The animal study was reviewed and approved by the Institutional Animal Care and Use Committees at The University of Texas MD Anderson Cancer Center.

## Author Contributions

HM designed, performed the experiments, and analyzed the data. HM and SG wrote the manuscript. LO assisted with the mouse study and performed experiments. SG, MPV, CB, MM, and AV provided materials and helped with the experimental design. MA and SG conceptualized the study, and designed experiments. MA, SG, and MPV provided funding. All the authors reviewed/edited the manuscript. All authors listed have made a substantial, direct, and intellectual contribution to the work and approved it for publication.

## Funding

This work was supported in part by research funding from the Paul and Mary Haas Chair in Genetics and the Cancer Prevention & Research Institute of Texas (CPRIT) grant RP130397 (to MA) and Leukemia & Lymphoma Society grant 6483-16 (to SG), the American Lebanese Syrian Associated Charities (to MV and SG). This work used MD Anderson Cancer Center Flow Cytometry and Cell Imaging, Research Animal Support, supported by the National Institutes of Health Cancer Center Support Grant (P30CA016672). The content is solely the responsibility of the authors and does not necessarily represent the official views of the NIH.

## Conflict of Interest

CB, MV, and SG have patent applications in the field of T-cell and gene-modified T-cell therapy for cancer. CB has received research funding from Merck, Sharpe, and Dohme, Inc., Bristol Myers Squibb and Kiadis Pharma. SG is a consultant for Catamaran Bio, Nektar Therapeutics, and TESSA Therapeutics, on the Scientific Advisory Board of Tidal, and a DSMB member of Immatics. MA is on the Scientific Advisory Board of Aptose and SentiBio and a consultant for Syndax and Glycomimetics.

The remaining authors declare that the research was conducted in the absence of any commercial or financial relationships that could be construed as a potential conflict of interest.

## Publisher’s Note

All claims expressed in this article are solely those of the authors and do not necessarily represent those of their affiliated organizations, or those of the publisher, the editors and the reviewers. Any product that may be evaluated in this article, or claim that may be made by its manufacturer, is not guaranteed or endorsed by the publisher.
