# Supplementary material for: Transgenic Expression of IL15 Retains CD123-Redirected T Cells in a Less Differentiated State Resulting in Improved Anti-AML Activity in Autologous AML PDX Models
Source: Front Immunol. 2022 May 9;13:880108. doi: 10.3389/fimmu.2022.880108 (PMC9124830; doi:10.3389/fimmu.2022.880108)
Supplement: Supplementary file 1 [file DataSheet_1.pdf]

Supplemental Figure S1

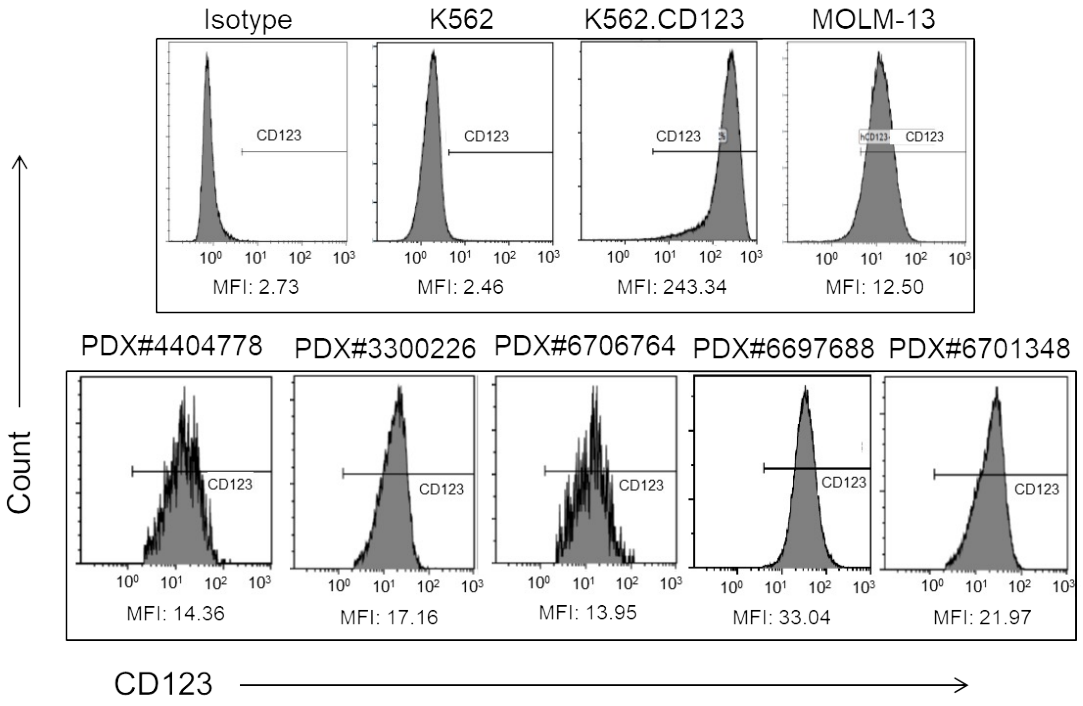

Supplemental Figure S1: CD123 expression in leukemia cell lines and AML patient samples by FACS. Representative plots and relative median florescence intensities (MFI) are shown. AML blast from PDX#4404778, PDX#3300226, and PDX#6706764 were used for *in vitro* studies shown in **Figure 1**. ENG T cells were generated from PDX#6697688, PDX#6701348, and PDX#6697688 for the studies shown in **Figure 5**.

Supplemental Figure S2

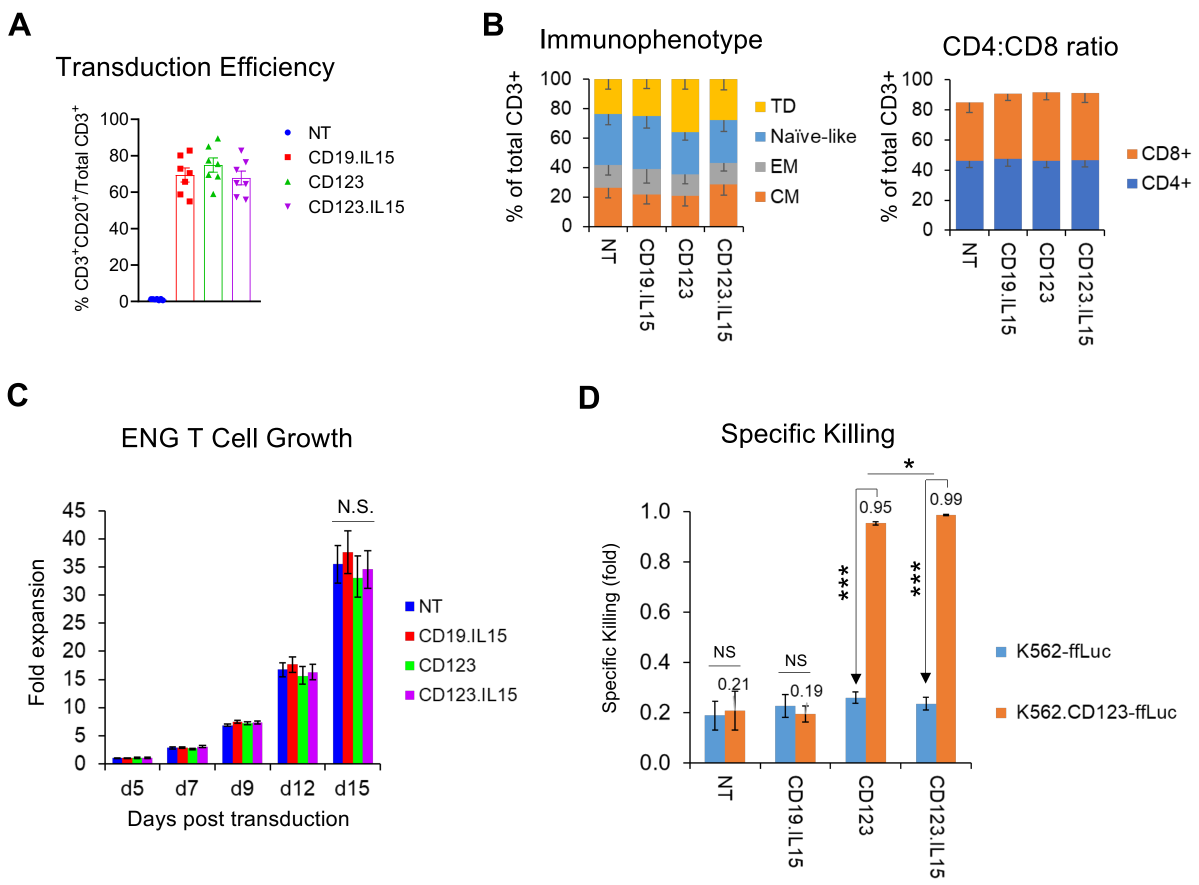

Supplemental Figure S2: Phenotypic and functional characterization of ENG T cells. (**A**) Transduction efficiency of engager T cells by FACS. Data is shown the frequency of $CD20^{+}CD3^{+}$/total live $CD3^{+}$ T cells. n=7 healthy donors. (**B**) Phenotypic analysis and $CD4^{+}$ to $CD8^{+}$ T-cell ratio of $CD20^{+}CD3^{+}$ T cells vs $CD3^{+}$ NT T-cells. A mixture of $CD4^{+}$ and $CD8^{+}$ T-cells and the presence of naïve: $CD45RA^{+}$/$CCR7^{+}$, central memory: $CD45RA^{+}$/$CCR7^{-}$, effector memory: $CD45RA^{-}$/$CCR7^{-}$, and terminally differentiated effector memory: $CD45RA^{-}$/$CCR7^{+}$, n=7. (**C**) T cell growth rate during *ex vivo* expansion during day 5 to day15 post transduction. Viable T-cells were measured using Vi-CELL XR cell counter and the growth rates were shown as fold change vs the cell number on the days of transduction. n=7. (**D**) Cytotoxicity by luciferase activity assay. T cells ($1.5x10^{6}$ cells/well) were co-cultured with firefly luciferase expressing K562.CD123 or K562 leukemia cells with E:T ratio of 3:1. After 20 hours, luciferase activity of live leukemia cells were measured, and specific killing is calculated based on total flux (p/s) and shown as 100*([p/s] of leukemia cells only control – [p/s] of experimental group)/([p/s] of leukemia cell only control). n=3, duplicate in assay. All data are shown as mean ± SE. ***p:<0.001, *p:<0.05, n.s.: not significant, 2-way ANOVA with Tukey's multiple comparison test.

Supplemental Figure S3

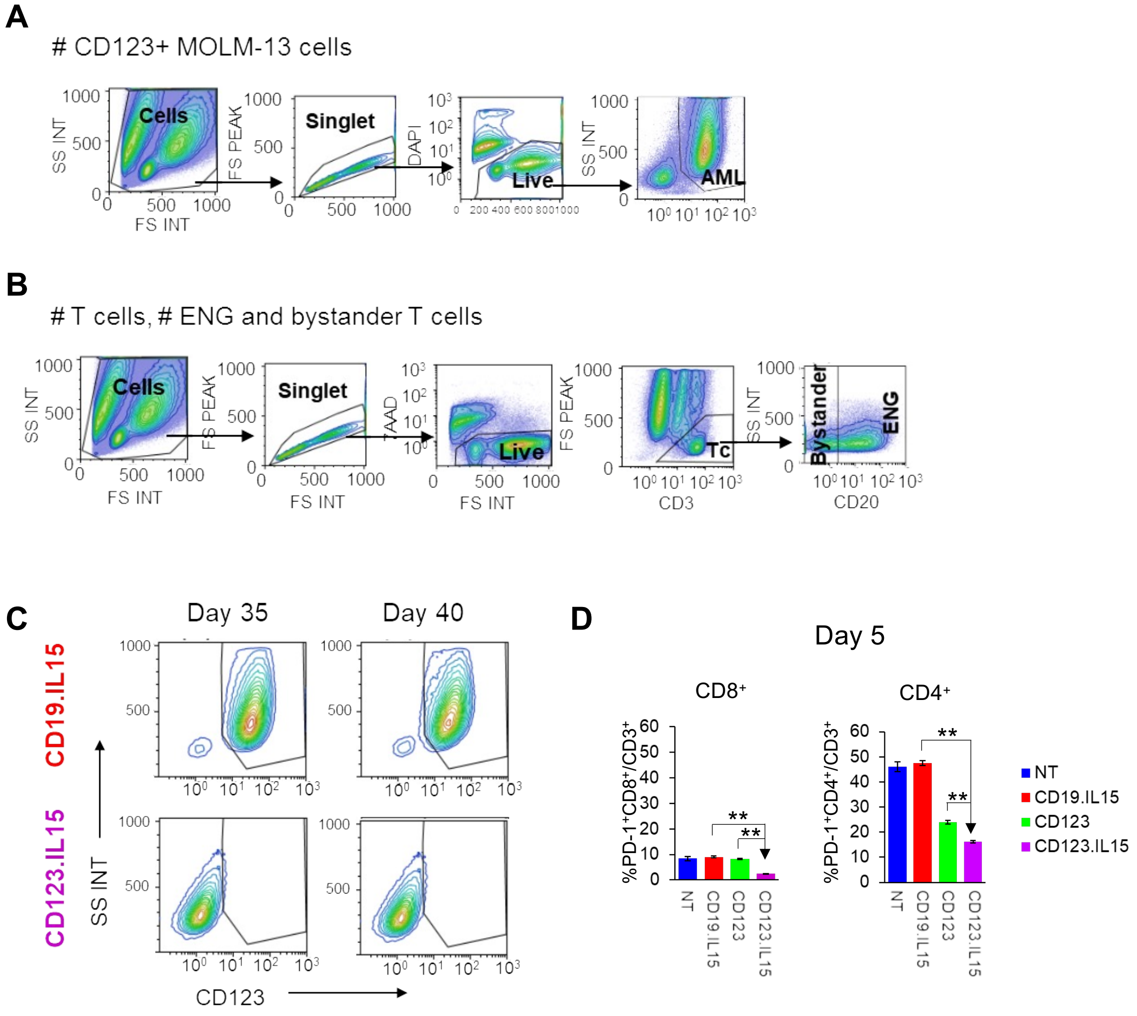

Supplemental Figure S3: CD123-ENG.IL15 T-cells have improved effector function. Supplementary data for the experiment shown in **Figure 2**. (**A**) Gating strategy on live CD123^{+} MOLM-13 AML cells in co-culture. (**B**) Gating strategy on live CD3^{+} T cells and CD3^{+}CD20^{+} engager and CD3^{+}CD20^{-} bystander T cells in co-culture. (**C**) Representative FACS plots showing live CD123^{+} MOLM-13 AML cells in co-culture on day 35 and day 40 over repetitive tumor challenge. **(D**) PD-1 and TIM3 expression in CD8^{+} and CD4^{+} T-cells on day 5 in coculture. Frequency of PD-1^{+}CD8^{+} and PD-1^{+}CD4^{+} in total live CD3^{+} T cells are shown. N=3. All values are shown as mean ± SE. ***:p<0.001, **:p<0.01, *:p<0.05, n.s.: not significant, *t*-test.

Supplemental Figure S4

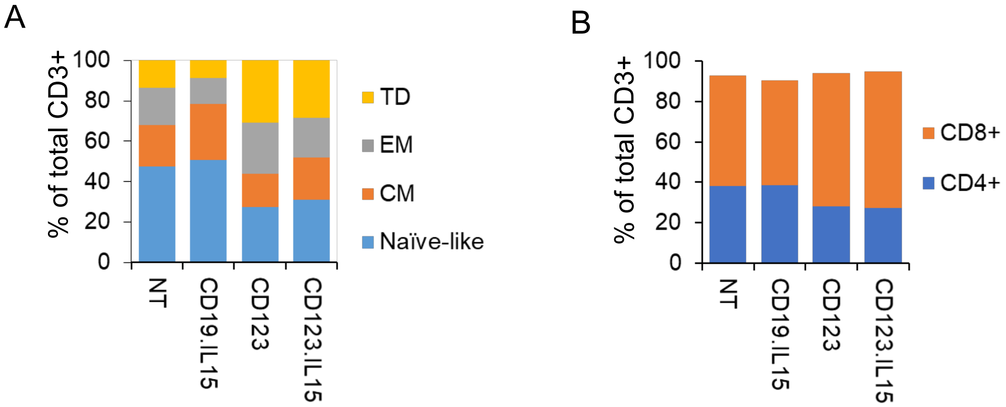

Supplemental Figure S4: Phenotype of CD123-ENG.IL15 T cells for MOLM-13 AML xenograft model. Phenotype of T-cell populations prior to infusion. (**A**) T-cell memory subsets. (**B**) CD4 and CD8 T-cell subsets.

Supplemental Figure S5

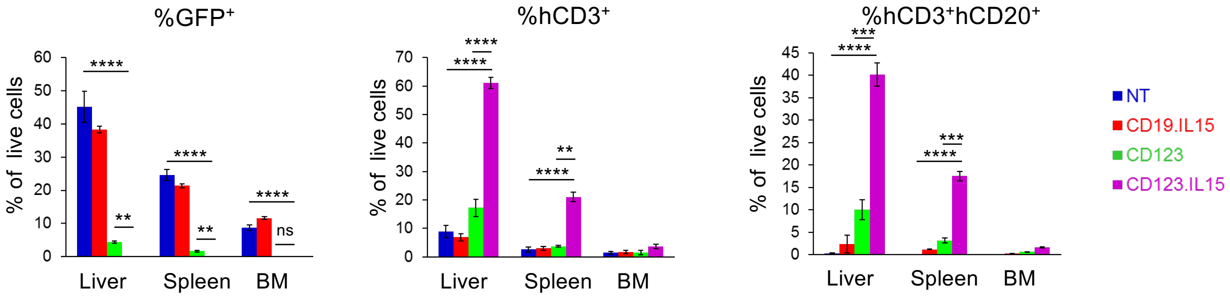

Supplemental Figure S5: CD123-ENG.IL15 T-cells have anti-AML activity and persist at higher frequency than CD123-ENG T cells in mouse tissues of MOLM-13 xenograft model. Supplementary data for the experiment shown in **Figure 4G**. **Figure 4G** shows normalized data to allow for comparison across liver, spleen, and bone marrow (BM). Here absolute values for each anatomic site is shown. Please see legend for **Figure 4G** for additional detail.

Supplemental Figure S6

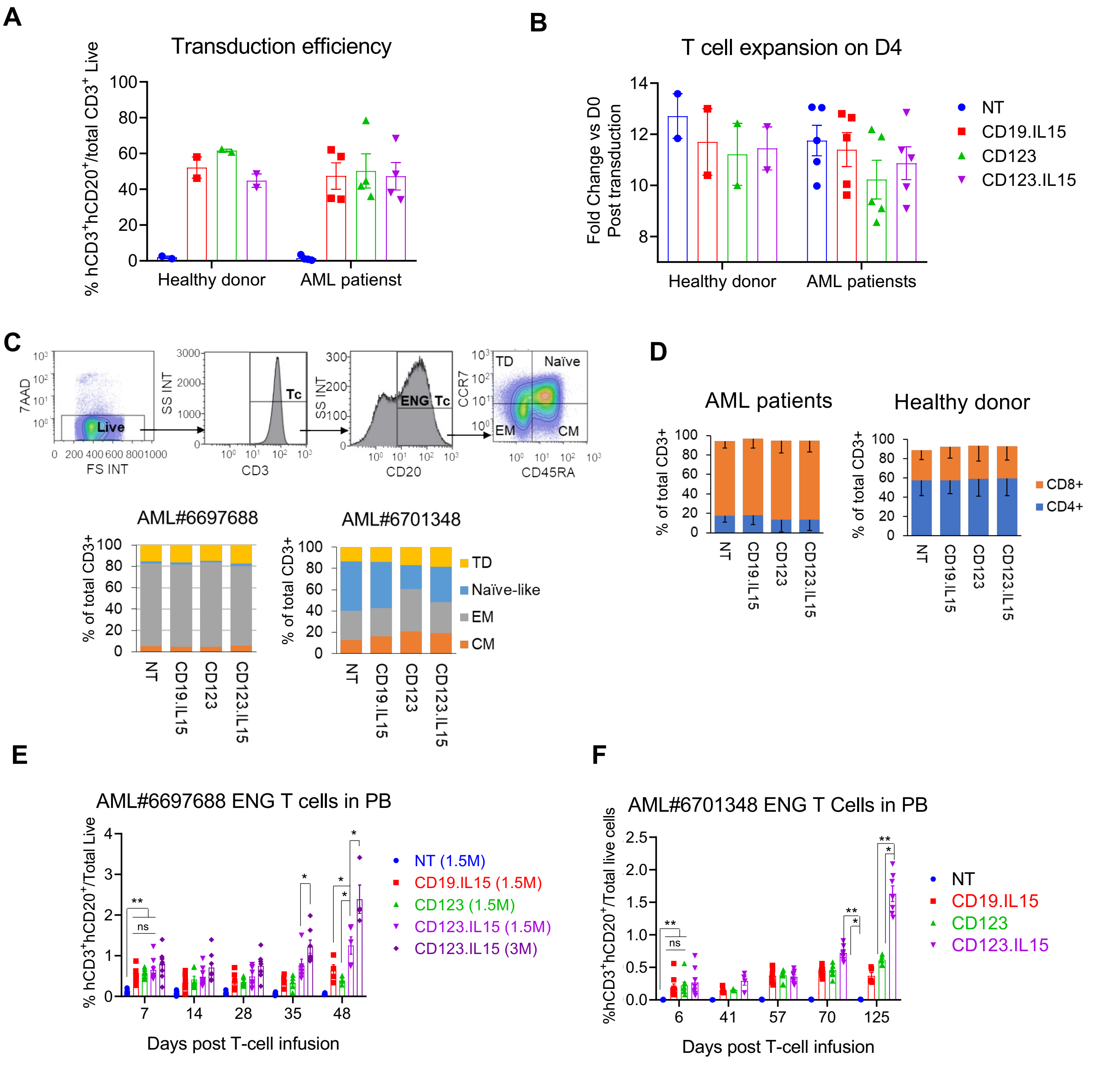

Supplemental Figure S6: CD123-ENG.IL15 T-cells have improved effector function in AML PDX NSGS mouse models. Supplementary data for the experiment shown in **Figure 4**. (**A**) Transduction efficiency of engager T cells by FACS. Data shows the frequency of hCD20$^+$hCD3$^+$/total live hCD3$^+$ T cells; n=3. (**B**) Comparison of T cell growth from healthy donors vs AML patients during *ex vivo* expansion. (**C**) Gating strategy for immune-phenotypic analysis by FACS (top). Phenotype of T-generated T-cell populations (bottom). (**D**) Phenotypic analysis of CD4$^+$ to CD8$^+$ T cells ratio of hCD20$^+$hCD3$^+$ T cells from healthy donors and AML patients; n=2 healthy donors; n=3 AML patients. Circulating hCD45$^+$hCD3$^+$hCD20$^+$mCD45$^-$ engager T-cells in total live cells of mouse PB in the AML (**E**) PDX#6698688 and (**F**) PDX#6701348 mouse models post T-cell infusion. Data shown as mean ± SE. ***:p<0.001, **:p<0.01, ns: not significant.

Supplemental Figure S7

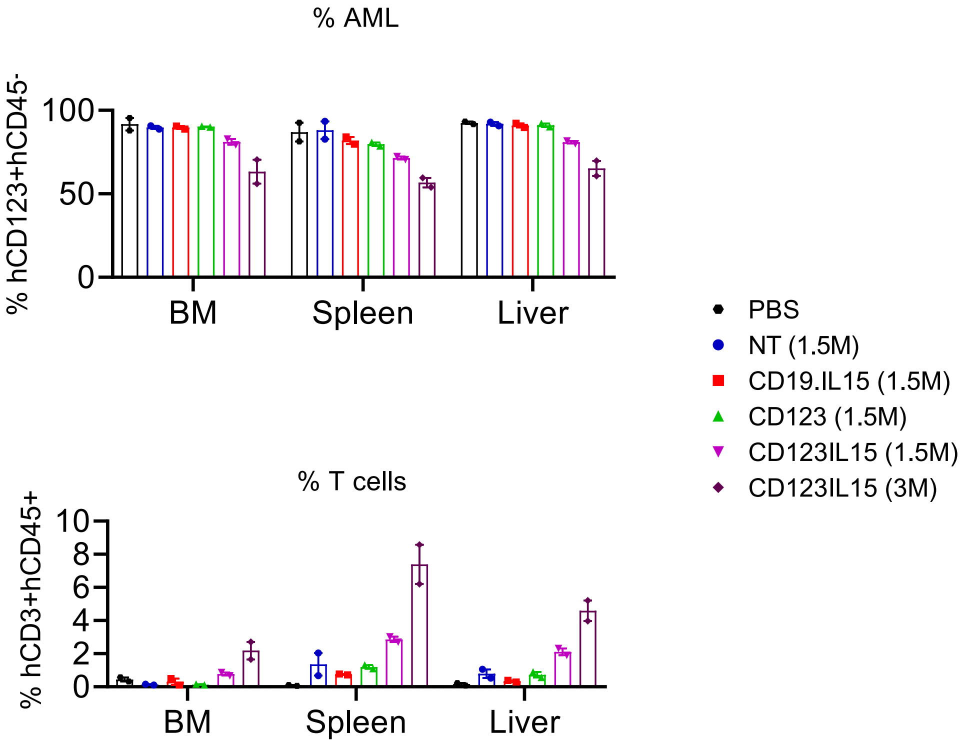

Supplemental Figure S7: CD123-ENG.IL15 T-cells have anti-AML activity and persist at higher frequency than CD123-ENG T cells in AML PDX#6697688. Supplementary data for the experiment shown in **Figure 5G**. **Figure 5G** shows normalized data to allow for comparison across liver, spleen, and bone marrow (BM). Here absolute values for each anatomic site is shown. Please see legend for **Figure 5G** for additional details.

Supplemental Figure S8

A.

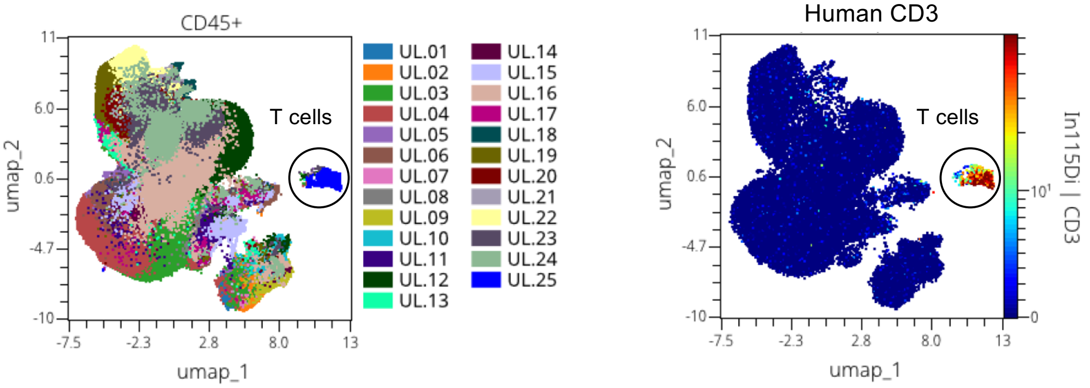

B.

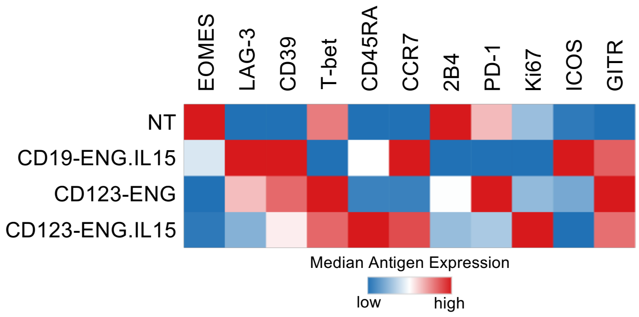

Supplemental Figure S8: CD123-ENG.IL15 T cells retain a less differentiated state in vivo and express lower levels of exhaustion markers. BM and spleen bulk cells from AML PDX#6697688 mice (day 28 post T cells infusion) were isolated and analyzed by CyTOF mass cytometry. (**A**) UMAP plot shows 25 clusters were identified in BM/spleen cells (total 4 samples overlaid, left); autologous T cells clustered in UL25 as judged by human CD3 expression (circled, right). (**B**) Human T cells were analyzed for T-cell exhaustion (EOMES, LAG-3, PD-1, TBET, 2B2, ICOS, CD39, and GITR), proliferation (Ki-67), and phenotype (CCR7 and CD45RA); data presented as heatmap.

Supplemental Figure S9

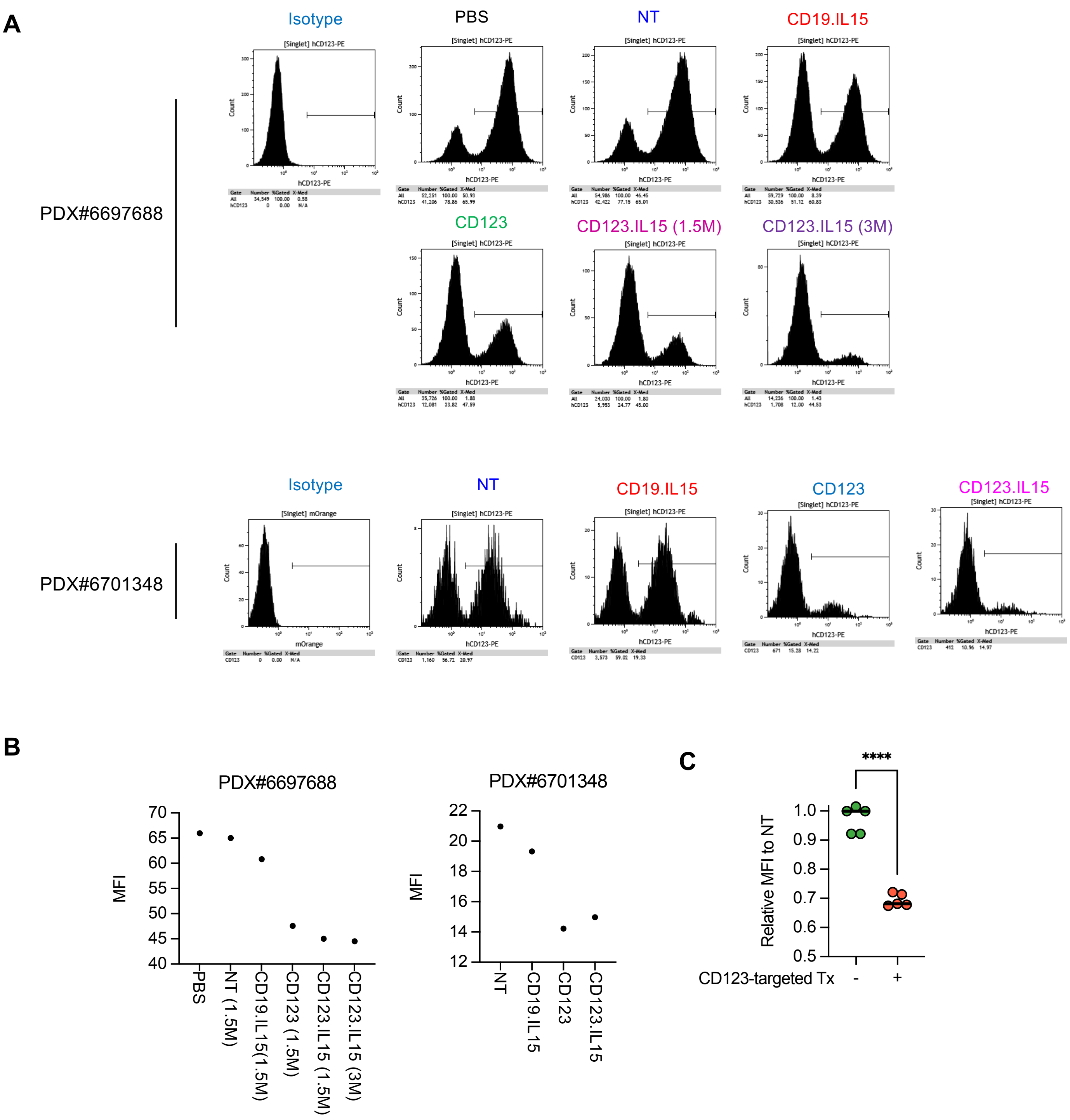

Supplemental Figure S9: Reduced CD123 antigen expression in circulating AML blasts in two AML PDX models under autologous T cell therapy. Peripheral blood samples of mice were obtained on day 27 (PDX#6697688) or day 125 (PDX#6701348) post T-cell infusion of experiment shown in Figure 4 and analyzed for the presence of human CD123+ AML cells by flowcytometry (n=1 per PDX model). (**A**) FACS plots. (**B**) Mean fluorescence intensity (MFI) of CD123 expression in AML blasts. (**C**) Normalized MFI to NT for each PDX. CD123-targeted (CD123, CD123.IL15) was compared to non-targeted (NT, PBS, CD19.IL15) therapy (Tx), ****:p<0.0001, *t*-test.
