# Supplementary material for: Transgenic Expression of IL15 Retains CD123-Redirected T Cells in a Less Differentiated State Resulting in Improved Anti-AML Activity in Autologous AML PDX Models
Source: Front Immunol. 2022 May 9;13:880108. doi: 10.3389/fimmu.2022.880108 (PMC9124830; doi:10.3389/fimmu.2022.880108)
Supplement: Supplementary file 2 [file DataSheet_2.pdf]

Table S1: List of antibodies used for multiplexed mass cytometry

Isotope	Metal	Antigen	Clone	Vendor	Category
89	Y	CD45	HI30	Biolegend	Barcoding
104	Pd	CD45	HI30	Biolegend	Barcoding
105	Pd	CD45	HI30	Biolegend	Barcoding
106	Pd	CD45	HI30	Biolegend	Barcoding
108	Pd	CD45	HI30	Biolegend	Barcoding
110	Pd	CD45	HI30	Biolegend	Barcoding
113	In	CD8	RPA-T8	Biolegend	Surface
115	In	CD3	HI30	Biolegend	Surface
127	I	N/A	N/A	Sigma-Aldrich	Cell cycle
141	Pr	CCR6	G034E3	Fluidigm	Surface
142	Nd	CD27	M-T271	BD	Surface
143	Nd	CD56	NCAM16.2	BD	Surface
144	Nd	CD26	BA5b	BioLegend	Surface
145	Nd	NKG2A	REA110	Miltenyi	Surface
146	Nd	CD49F	GoH3	BD	Surface
147	Sm	CD127	REA614	Miltenyi	Surface
148	Nd	CCR2	REA264	Miltenyi	Surface
149	Sm	CCR4	205410	R&D	Surface
150	Nd	CD28	CD28.2	BioLegend	Surface
151	Eu	T-bet	O4-46	BD	Intracellular
152	Sm	TIGIT	MBSA43	Thermofisher	Surface
153	Eu	CD45RA	HI100	Biolegend	Surface
154	Sm	Ki-67	20Raj1	Thermofisher	Intracellular
155	Gd	CCR7	G043H7	Biolegend	Surface
156	Gd	TIM3	F38-2E2	Biolegend	Surface
158	Gd	2B4	C1.7	Biolegend	Surface
159	Tb	ICOS	C398.4A	Biolegend	Surface
160	Gd	PD-1	EH12.2H7	BD	Surface
161	Dy	CXCR3	G025H7	Biolegend	Surface
162	Dy	Eomes	WD1928	Thermofisher	Intracellular
163	Dy	CD137	4B4-1	BD	Surface
164	Dy	CD20	2H7	Biolegend	Surface
165	Ho	CD25	2A3	BD	Surface
166	Er	LAG3	REA351	Miltenyi	Surface
167	Er	CXCR5	J252D4	Biolegend	Surface
168	Er	CD160	688327	R&D	Surface
169	Tm	CD69	FN50	BD	Surface
170	Er	CD39	A1	Bio-Rad	Surface
171	Yb	CD161	191B8	Miltenyi	Surface
172	Yb	KLRG1	13F12F2	Thermofisher	Surface
173	Yb	CD95	DX2	BD	Surface
174	Yb	GITR	DT5D3	Miltenyi	Surface
175	Lu	CD4	RPA-T4	Biolegend	Surface
176	Yb	CD38	REA572	Miltenyi	Surface
191	Ir	DNA	N/A	Fluidigm	Intercalator
193	Ir	DNA	N/A	Fluidigm	Intercalator
194	Pt	CD57	HCD57	Biolegend	Surface
195	Pt	N/A	N/A	Enzo	Live-dead
196	Pt	N/A	N/A	Enzo	Live-dead
198	Pt	HLA-DR	L243	Biolegend	Surface
209	Bi	CLA	HECA-452	BD	Surface
